# Experimental study of perturbative particle transport in the HL-2A tokamak

**DOI:** 10.1016/j.heliyon.2020.e04633

**Published:** 2020-09-14

**Authors:** B.Y. Zhang, S. Inagaki, Z.B. Shi, W.L. Zhong, X.L. Zou, M. Jiang, Z.C. Yang, P.W. Shi, C.Y. Chen, G.L. Xiao, B.B. Feng, X.M. Song

**Affiliations:** a Research Institute for Applied Mechanics, Kyushu University, Japan; b Southwestern Institute of Physics, China; c The French Alternative Energies and Atomic Energy Commission, France

**Keywords:** Nuclear physics, Plasma physics, Tokamak, Plasma, Particle transport, Supersonic molecular beam injection

## Abstract

Perturbative particle transport experiment has been performed in the HL-2A tokamak by using supersonic molecular beam injection (SMBI) as an external particle source. The spatiotemporal evolution of edge density perturbation is traced and the particle source and the flux-gradient relation are obtained experimentally. The flux-gradient relation is found to be far from the diffusive model and three different transport processes are revealed, including pinch-dominant process, diffusion-pinch process and intermittent decays.

## 1 Introduction

For the realization of magnetic fusion reactor, achievement of dense core plasma is necessary [1]. Particle transport in tokamaks and helical devices has been, however, an unresolved mystery for over 30 years. It was found to be difficult to explain radial density profile by diffusive transport models [2, 3]. A turbulent pinch is proposed to explain the profile peaking without central fueling [4]. Experimental observations indicate the connection between density peaking and turbulent state, which is predicted by turbulent transport theory [5]. Although the pinch model is consistent with experimental trends, there still are many questions remaining unsolved which may arise from lack of understanding of turbulence. Recently, perturbative method has been proved to be one of the most effective ways to study particle transport in plasma. Particle transport coefficients, i.e. diffusivity and convection velocity are determined [6, 7, 8], and some progress on transport barriers and transport mechanism has been achieved [9, 10, 11]. To confirm the non-diffusive nature of particle transport associated with the turbulent pinch, perturbative particle transport experiment has been performed in the HL-2A tokamak with supersonic molecular beam injection (SMBI), and microwave reflectometers [12] are used as the main diagnostic system to study particle transport.

## 2 Experimental setup

**Figure 1:**
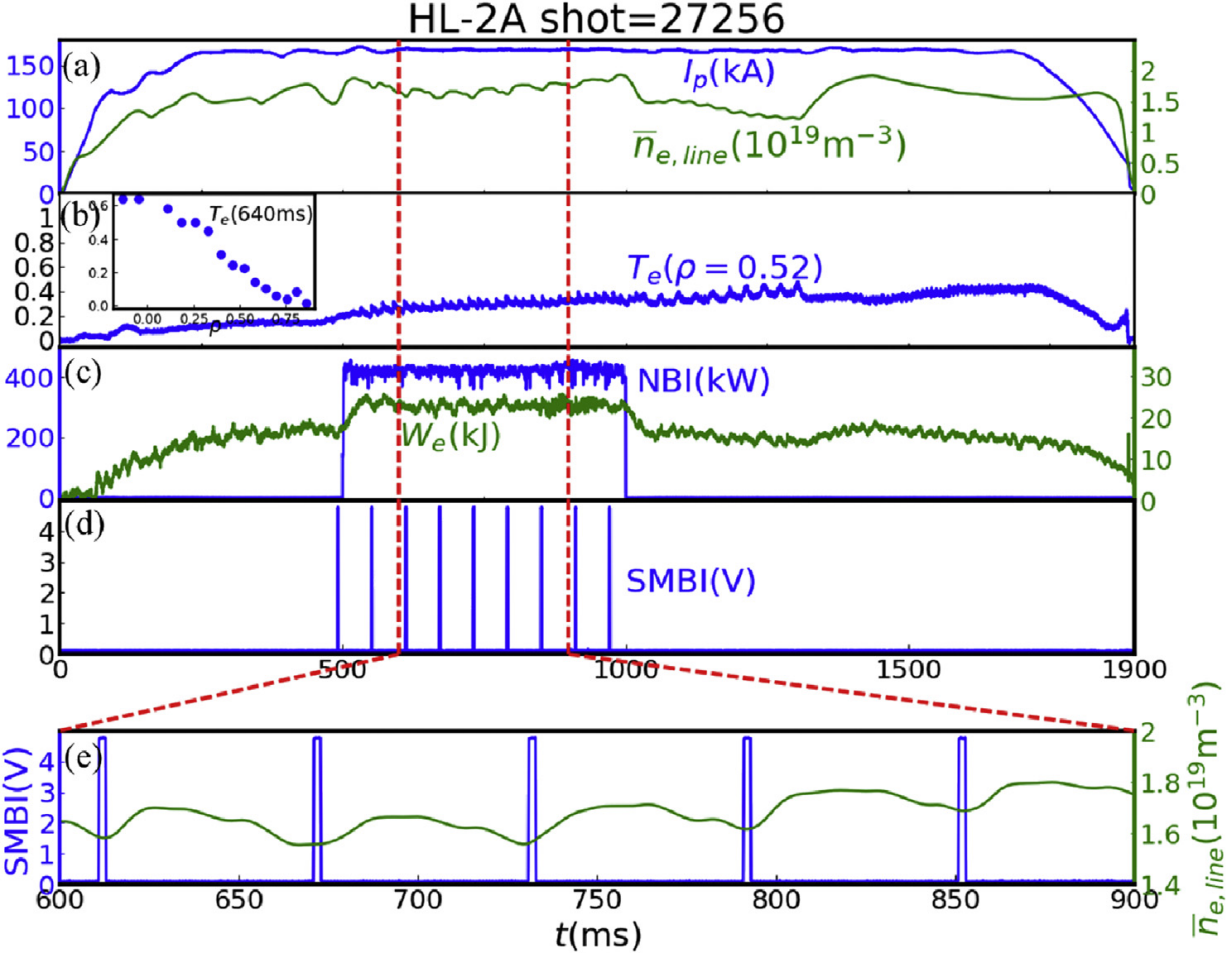
The temporal evolution of (a) plasma current, line-average density, (b) electron temperature, (c) NBI power, stored energy and (d) SMBI signal in the particle transport experiment. (e) is the enlarged view for density and SMBI.

A perturbative particle transport experiment has been performed in the HL-2A tokamak [13]. The discharge is performed in L-mode, and the plasma is circular with divert configuration. The parameters in this discharge are as follows: major radius R=1.65m, minor radius a=0.4m, magnetic field $B_{0}=1.35\;\text{T}$, plasma current $I_{p}=170\;\text{kA}$, and background electron density $n_{e}=1.4\times 10^{19}\;{\text{m}}^{-3}$. Figure 1 shows the temporal evolution of plasma parameters of a typical discharge, where the electron temperature $T_{e}$ is measured at normalized radius ρ=r/a=0.52 with an electron cyclotron emission (ECE) radiometer [14] and the line-average density ${\bar{n}}_{e,\;line}$ is measured with a far infrared rays (FIR) laser interferometer [15]. Plasma current $I_{p}$ is kept stable from 200 ms to 1700 ms. Neutral beam (NBI, 400 kW, co-) is injected from 500 ms to 1000 ms, during which the stored energy $W_{e}$ is obviously increased. The SMBI, which has the advantages of deep penetration and high fueling efficiency [16], is used as perturbative particle source. Electron density is modulated by periodical SMBI (repetition period of 60 ms and pulse width of 2 ms) during NBI heating phase as shown in Figure 1.

**Figure 2:**
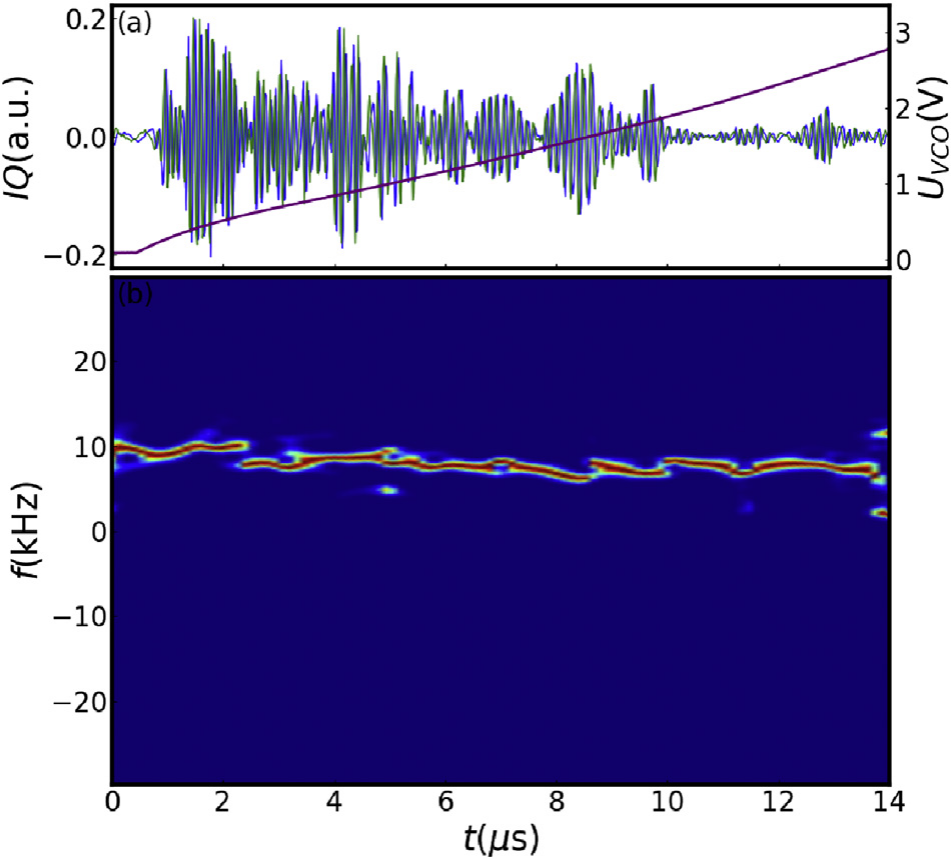
(a) The initial IQ signals and (b) the time-frequency spectrum of reflected wave of microwave reflectometer.

A sweeping frequency microwave reflectometer with high temporal resolution (up to 6 μs for a frequency sweep) is used to measure the density profile and trace perturbation [17]. A dynamic calibration method of voltage controlled oscillator (VCO) is developed and dispersion of the transmission system is taken into account to reduce measurement error [17]. Figure 2 shows the director signal from the IQ (in-phase and quadrature phase) detector and the temporal evolution of beat frequency extracted from the spectrogram in a sweeping period. The beat frequency keeps mostly stable except for some frequency jump points and thus phase delay is obtained according to this. The phase losses at frequency jump points are modified with numerical method [18].

## 3 Experimental results

**Figure 3:**
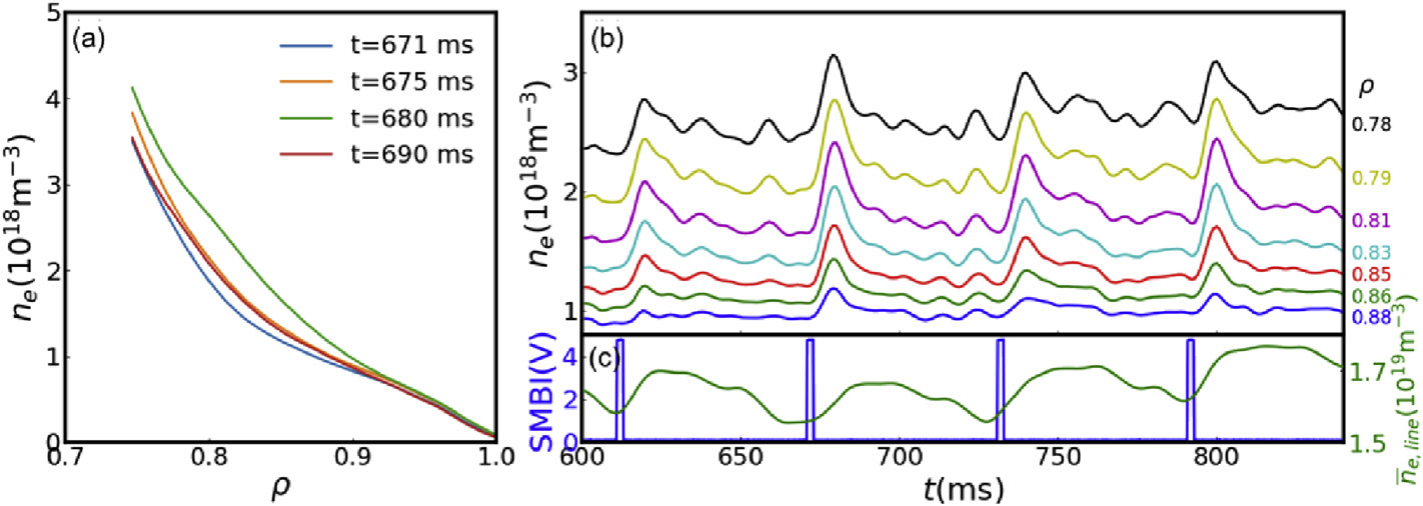
(a) Radial profile of density. (b) Temporal evolution of local density. (c) SMBI signal and line-average densit.

The radial profiles and temporal evolution of edge electron density are reconstructed with the reflectometer, which are shown in Figure 3(a) and (b), respectively. It is clear that the edge density rapidly responses to SMBI fueling and is strongly modulated. The line-average density measured by FIR at ρ=0.1, where ρ=r/a is the normalized radius, is shown as the green line in Figure 3(b) and is modulated by the SMBI as well. The perturbative particle transport can thus be studied from the density perturbation.

### 3.1 Evaluation of transport coefficients

**Figure 4:**
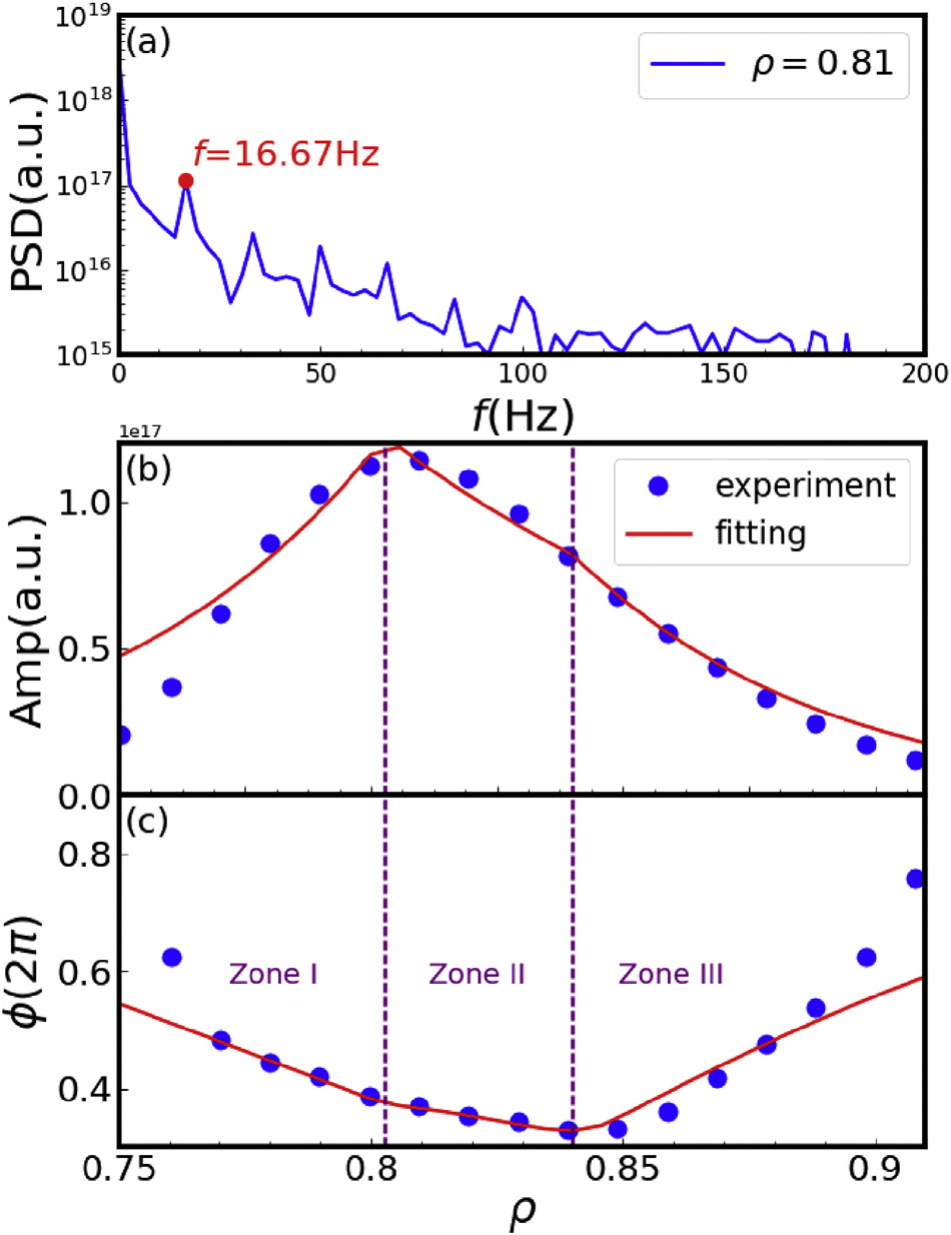
(a) Frequency spectrum of density perturbation at ρ=0.81. Profiles of (b) amplitude and (c) phase of density perturbation at the modulation frequency.

For periodic density perturbation, it is possible to evaluate the transport parameters (e.g. diffusivity D and convective velocity V) from the amplitude and phase of the density perturbation, which are given by the FFT analysis [11, 19]. As shown in Figure 4(a), the first harmonic frequency of density perturbation at ρ=0.81 is 16.67 Hz, which corresponds to the modulation frequency of SMBI. The amplitude and phase of the density perturbation at the modulation frequency is thus calculated, and the whole profiles are plotted as the blue dots show in Figure 4(b) and (c). It is obvious that the minimum of the phase profile is located at around ρ=0.84. This is corresponding to the initial position of the modulated density perturbation. It is also noted that the peak of the amplitude profile, indicating the maximum density perturbation and the main particle source deposition, is located at around ρ=0.8. The plasma edge is thus divided into three zones according to the sharp change of amplitude and phase profiles, as shown in Table 1.

**Table 1:** Diffusivity and convective velocity in different zones.

	Zone I	Zone II	Zone III
ρ	0.75<ρ<0.802	0.802<ρ<0.84	0.84<ρ<1
$D\;\left({\text{m}}^{2}/s\right)$	0.2	0.7	0.11
V(m/s)	10	-22	-6

As shown in Ref. [20], the perturbation phase is mainly dominated by the diffusivity, while the amplitude depends on both the diffusivity and the convective velocity. The simulation fitting of the amplitude and phase profiles can thus be achieved by separately determining the transport parameters in each zone with an analytical linear transport model [19]. The source is assumed to be Gaussian-like with the half-width simulated and the peak located at the initial position of the modulated density perturbation (i.e., the location of the minimum of the phase profile in Figure 4(c)). The strength of the source is set according to the pressure and duration of the SMBI. The red lines in Figure 4(b) and (c) show the best fit to the amplitude and phase profiles with the optimized D and V listed in Table 1. The negative and positive values of V indicate the inward and outward convection, respectively. It is noted that a significant inward pinch exists in Zone II, which may contribute to the inward shift of particle deposition and plays a crucial role for the fueling.

However, this model presumes a diffusive-like flux-gradient relation, which is not confirmed yet. The flux-gradient relationship thus should be directly analyzed and be compared with the simulation result to confirm whether flux is proportional to gradient and whether turbulent pinch exists. In addition, transport parameters will be changed depending on the modeling of spatiotemporal structure of particle source. The modeling of particle source of SMBI has not been established yet. Sometimes the SMBI even consists of a fast component and a slow component, and penetration depth and time-to-peak (at which particle production rate becomes maximum) of them are different [21]. This makes modeling more difficult.

### 3.2 Particle source

**Figure 5:**
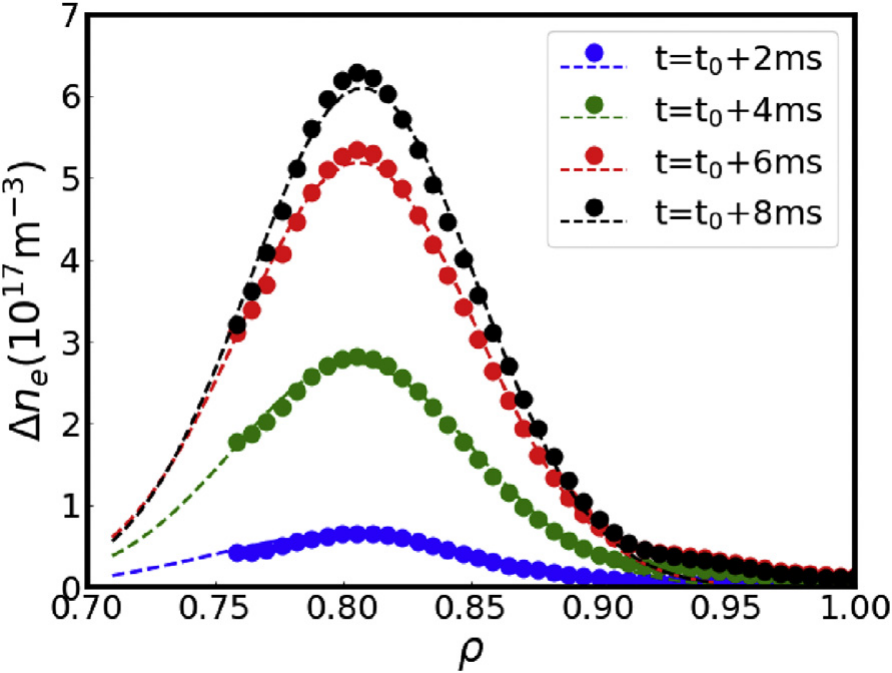
Temporal evolution of density perturbation in the early phase of SMBI.

The determination of the particle source is essential in perturbative particle transport study. To estimate the location of particle source experimentally, the density perturbation after SMBI is calculated as $\Delta n_{e}=n_{e}\left(t_{0}+\Delta t\right)-n_{e}\left(t_{0}\right)$, where $t_{0}$ is the starting time of SMBI and Δt is the time delay after SMBI. The particle source can be estimated by $\Delta n_{e}$ while Δt is short enough, which means the transport is negligibly small. Figure 5 illustrates the temporal evolution of the radial profile of density perturbation shortly after SMBI. The dots in Figure 5 are the experimental measurements of density perturbations at 2 ms, 4 ms, 6 ms and 8 ms after SMBI respectively, and the dashed lines are the relevant Gaussian fittings. It is noted that the profiles are the averaged results over 4 SMBIs which are shown in Figure 3(b). Figure 5 clearly shows that the particle source is Gaussian like and is deposited at around ρ=0.81, which agrees well with the maximum of the amplitude profile in Figure 4(b).

**Figure 6:**
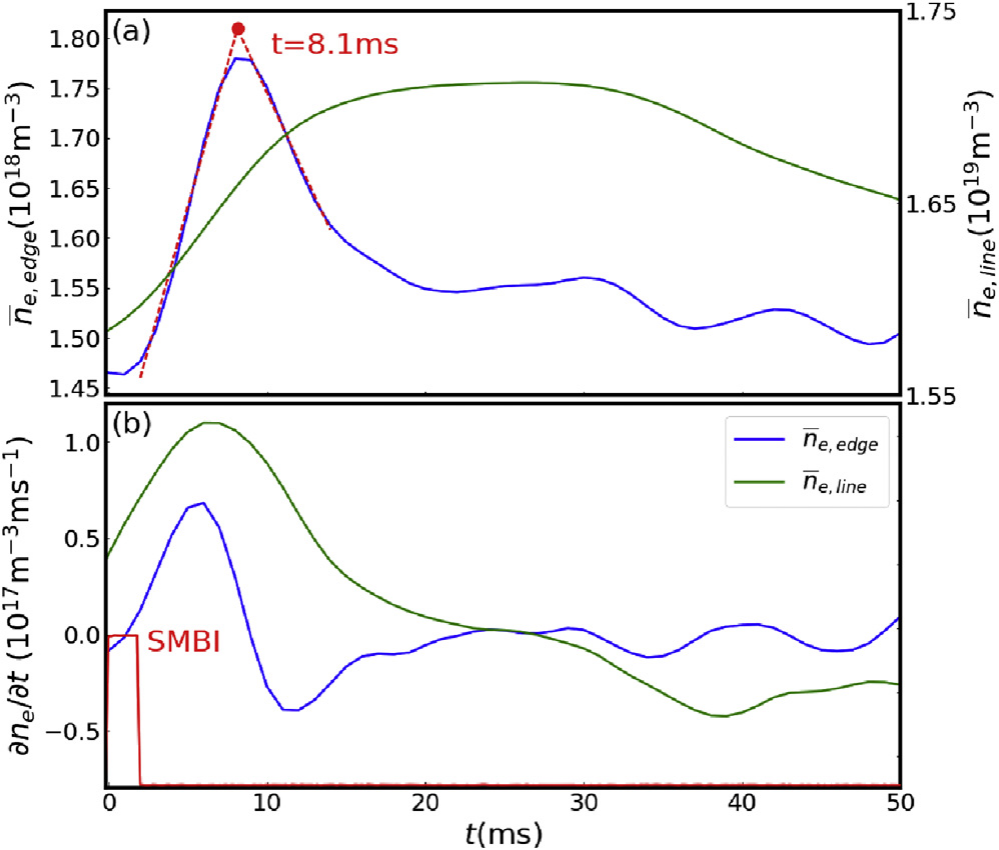
(a) Temporal evolution and (b) temporal derivative of edge density and line-average density after SMBI.

Particle source has a direct influence on spatiotemporal behaviors of electron density perturbation, then spatiotemporal region of source should be distinguished from source-free region. It is difficult to distinguish and separate the source-free region in the FFT analysis thus we re-considered the temporal behaviors of density with or without edge particle source. Figure 6 displays the temporal evolution and temporal derivative of edge-average density ${\bar{n}}_{e,\;edge}$ (defined as the line average of edge density profile for ρ>0.75) and line-average density after SMBI, which is averaged for the 4 SMBIs shown in Figure 3(b) (assumed to be injected at 0 ms). It shows that just after the SMBI, edge-average density rapidly increases from approximately $1.45\times 10^{18}{\text{m}}^{-3}$ to $1.75\times 10^{18}{\text{m}}^{-3}$, and reaches the maximum at around 8 ms. Meanwhile, line-average density increases slowly. A linear fitting of edge-average density evolution also shows that the turning point of density growth is located at 8.1 ms, which indicates that the SMBI source is fully ionized and the transport is source-free since then. Before 8 ms, temporal evolution of density is dominated by particle source.

During the source phase, the growth rate of edge-average density increases rapidly in 5 ms and then returns to zero in 3 ms. In contrast, the line-average density always keeps increasing slowly, and the growth rate reaches the maximum at around 8 ms. This phenomenon indicates that the particle source induced by SMBI deposits at the edge and transports to the core region, resulting to the rapid growth of edge density and delayed growth of central density during the source phase.

### 3.3 Core transport

During the source-free phase from 8 ms after SMBI, core and edge regions show different transport characteristics. In Figure 6 it is shown that while edge density starts to decrease, the core density, which is represented by the line-average density, still keeps growing. The growth of the core density continues until 15 ms when the growth rate is almost zero. This indicates that there is a strong inward flux from edge to core, which is likely to be inward pinch. During this phase, the density profile should be steepened. From 15 ms to 30 ms, the core density remains almost constant while change in edge density is also small. This suggests that the steep profile stays unchanged due to zero particle flux, i.e. inward flux is balanced by outward flux which may be driven by the enhanced density gradient. After 30 ms, the core density starts to decrease, then the total flux seems to become outward. During this phase, the inward flux is considered to be suppressed.

### 3.4 Edge transport

**Figure 7:**
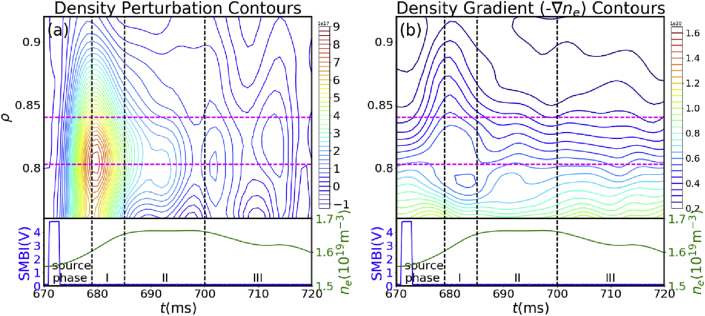
Contours of (a) density perturbation and (b) density gradient.

To evaluate the edge transport, the edge density perturbation $\left(\Delta n_{e}\left(t\right)=n_{e}\left(t\right)-n_{e}\left(t_{0}\right)\right)$ contours and edge density gradient contours are plotted as shown in Figure 7, where the dashed horizontal lines separate three transport zones corresponding to those in Figure 4. Here we show the transport process after SMBI at 671 ms as an example, and the transport phenomenon is similar for other SMBIs. It is clear that the edge density evolution experiences three different phases, which is consistent with core transport phases. When the core density still keeps increasing in phase I (from 8 ms to 15 ms after SMBI), the edge density decreases rapidly, which again demonstrates that a strong inward particle flux from the edge to the core region exists during this phase. Presence of inward flux at the edge is discussed later. It should be noted that during this phase, the density gradient at the edge almost remains constant, indicating that the inward flux may be driven by pinch. When the core density stops increasing and keeps almost stable in phase II (from 15 ms to 30 ms after SMBI), the edge density also slows down decreasing. Meanwhile, the density gradient gradually decays at this time. After 30 ms in phase III, the edge density shows some intermittent decays just before the decrease in the core density.

In order to reveal the transport process better, the flux-gradient relationship is required. The flux at radius $r_{0}$ is evaluated from time-dependent particle balance equation and written as

(1) $$-\text{Γ}=\frac{1}{S\left(r_{0}\right)}{∭}_{\text{Ω}\left(r\leq r_{0}\right)}\frac{\partial n_{e}}{\partial t}dV=\frac{1}{r_{0}}\int_{0}^{r_{0}}\frac{\partial n_{e}}{\partial t}rdr,$$

where $S\left(r_{0}\right)$ is the area of the flux plane at $r=r_{0}$ and Ω is the plasma volume. As the particle source perturbation induced by SMBI and the change of recycling is separated spatially and temporally, the particle source perturbation is neglected. Because of the limited waveband, the reflectometer can only cover part of the radial density profile. Thereby, to estimate the particle flux, the radial profile is divided into edge part and core part as below

(2) $$-\text{Γ}=\frac{1}{r_{0}}\int_{0}^{r_{1}}\frac{\partial n_{e}}{\partial t}rdr+\frac{1}{r_{0}}\int_{r_{1}}^{r}\frac{\partial n_{e}}{\partial t}rdr,$$

where $r_{1}$ is the innermost radius that the reflectometer covers. As the density profile and its temporal evolution at core region (i.e., the region where $r<r_{1}$) is unknown, a quadratic-like core density profile is assumed according to the interferometer diagnostic system [22].

(3) $$n_{e}\left(r\right)=n_{e}\left(0\right)\left[1-{\left(\frac{r}{r_{1}}\right)}^{2}\right]e^{-{\left(r/r_{1}\right)}^{2}}+n_{e}\left(r_{1}\right),$$

where $n_{e}\left(r\right)$ is the electron density at radius r ($r<r_{1}$). The core density calculated from this model is a function of $n_{\text{e}}\left(0\right)$, which is obtained experimentally from the line-average density by eliminating contribution of edge density. Thus, the core density profile and flux are obtained.

**Figure 8:**
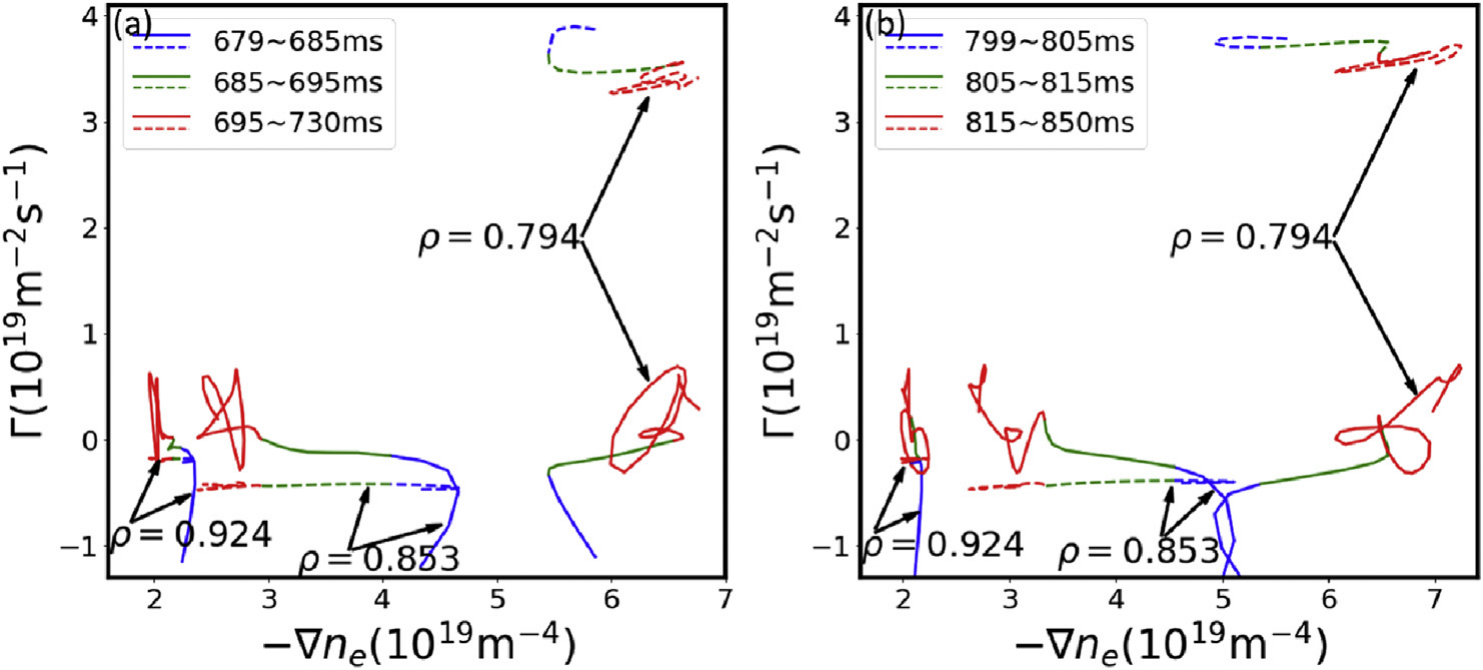
Experimental (solid lines) and simulative (dashed lines) flux-gradient dependences at different radii after the SMBI at (a) 670 ms and (b) 790 ms.

According to the formula above, the dependences of flux and gradient at different radial positions are given as the solid lines in Figure 8. Figure 8(a) and (b) are the transport processes for SMBIs at 670 ms and 790 ms, respectively. Similarly, these two figures both obviously show three different transport processes. Firstly, from 8 ms to approximately 15 ms after SMBI, the transport is dominated by inward flux, resulting to the decrease of edge density and increase of core density. The inward flux decreases rapidly during this phase while the gradient remains almost unchanged. This indicates that the inward flux is not driven by local density gradient. Instead, it is dominant by rapidly decreasing inward pinch and the inward pinch decreases rapidly. From approximately 15 ms–25 ms, the flux is close to zero, indicating that the diffusion and the inward pinch are comparable. The density gradient during this process significantly reduces outside of ρ=0.8, and the flux-gradient relation is close to linear, which seems to be a diffusive process but with a negative slope ($-\partial \text{Γ}/\partial \nabla n_{\text{e}}<0$). It is also noted that the inward flux slows down decreasing, which might result from the decrease of diffusion due to the relaxation of density gradient while the inward pinch keeps decreasing. On the contrary, the density gradient starts increase during this process in the region 0.75<ρ<0.8. This may result from the relaxation of particle source at source region (ρ=0.81) and the stability of density at the core region (ρ<0.75) due to the inward pinch. After 25 ms the intermittent outward flux is dominant while the density gradient is almost consistent, leading to the sudden decrease of both the edge and the core density. This indicates that the intermittent outward convection might be dominant during this process. A plausible conclusion is that the convection, which significantly varies during the whole transport process, greatly contributes to the perturbative particle transport.

It is also noted that the flux-gradient dependence from simulation (calculated via $\text{Γ}=-D\nabla n_{\text{e}}+Vn_{\text{e}}$ with the transport parameters listed in Table 1), which is shown as the dashed lines in Figure 8, is far from this picture. The flux is inward outside ρ=0.8 and outward inside ρ=0.8, respectively, during the whole transport process, and the variation of the inward flux is relatively small, which is against with the new analysis where the particle source is evaluated experimentally. The simulation results are qualitatively different from ones of the new analysis. A possible reason is that the transport parameters used in the simulation are quantitatively affected by the modeling of particle source, which has not been established yet. Another possibility is that the transport parameters are assumed to be time-independent, which cannot reflect the time-dependent transport processes shown in the new analysis, especially the dramatically variable convection. Therefore, even though the simulative amplitude and phase fit well with the experimental results in Figure 4(b) and (c), the transport parameters obtained via this method require to be improved.

### 3.5 Discussion

The first term of the right-hand side of Eq. (2) may have errors due to the lack of the local measurement of core density. However, increment of the core average-density and reduction of the edge density during source-free region clearly indicate the presence of inward flux. Therefore, flux-gradient relation shown in Figure 8 captures particle transport features of this experiment qualitatively.

Inhomogeneities of magnetic field and temperature are considered to cause turbulent pinch in tokamak [4]. Magnetic configuration is unchanged in the perturbative experiment and thus observed inward flux is not driven by magnetic field gradient. Concerning temperature gradient, there are no significant changes in the central ECE signal and then temperature gradient perturbation in the core may be negligibly small. However, since the edge plasma is optically thin, which results in the large error of the edge electron temperature measured with ECE, the accurate edge electron temperature profiles and evolution data are still missing. The evaluation of flux-temperature-gradient relation is left in the future work.

## 4 Summary

Particle transport is studied by perturbative experiment by using SMBI in HL-2A tokamak. Spatiotemporal evolution of edge density perturbation traced with a reflectometer with high temporal and spatial resolution allows us to estimate particle source and flux-gradient relation. This analysis indicates that i) inward particle pinch, which doesn't depend on the local gradient, is dominant shortly after the SMBI and rapidly decreases, ii) the diffusion decreases, along with the relaxation and increase of density gradients outside and inside of the source region, respectively, while the inward pinch is comparable and iii) intermittent process enhances outward flux at the edge. Even though the transport parameters given by the simulation with diffusive model fit well with the density perturbation, the flux-gradient relation obtained by the new analysis where the particle source is evaluated experimentally is far from the diffusive nature, indicating that more improvements are required for the time-independent diffusive model. An understanding of turbulence-driven flux will have deep impact on our predictability of evolution of density profile, and provide a sophisticated particle control knob of burning plasmas, such as ITER.

## Declarations

### Author contribution statement

B. Y. Zhang: Conceived and designed the experiments; Performed the experiments; Analyzed and interpreted the data; Contributed reagents, materials, analysis tools or data; Wrote the paper.

S. Inagaki: Analyzed and interpreted the data; Wrote the paper.

Z. B. Shi, W. L. Zhong, X. L. Zou: Conceived and designed the experiments; Contributed reagents, materials, analysis tools or data.

M. Jiang, Z. C. Yang, P. W. Shi, B. B. Feng: Performed the experiments.

C. Y. Chen, G. L. Xiao, X. M. Song: Performed the experiments; Contributed reagents, materials, analysis tools or data.

### Funding statement

This work was supported by 10.13039/501100012166National Key R&D Program of China (Grant Nos. 2017YFE0301203 and 2017YFE0301106), 10.13039/100012542Sichuan Science and Technology Program (Grant No. 2018RZ0123) and 10.13039/501100001809National Natural Science Foundation of China (Grant No. 11475057) and the Grant-in-Aid for Scientific Research of JSPF, Japan (15H02335, 17H06089).

### Competing interest statement

The authors declare no conflict of interest.

### Additional information

No additional information is available for this paper.
